# European Public Health News

**DOI:** 10.1093/eurpub/ckac157

**Published:** 2022-11-30

**Authors:** Maaike Droogers, Marie Guichardon, Marieke Verschuuren, Stella Kyriakides, Hans Henri P Kluge, Jarno Habicht, Ledia Lazeri, Alisa Ladyk-Bryzgalova, Roy Wadia, Rayyan Sabet-Parry, Alona Roshchenko, Jason Maurer, Anthony Staines, Floris Barnhoorn

**Affiliations:** (EUPHA); (EUPHA); (EUPHA); (European Commission); (WHO Regional Office for Europe); (WHO Ukraine); (WHO Regional Office for Europe); (WHO Ukraine); (WHO Regional Office for Europe); (WHO Ukraine); (WHO Ukraine); (WHO Regional Office for Europe); (EPH Conference); (EPH Conference)

At the time of writing of this editorial, the 15th EPH Conference that will take place in Berlin from 9 to 12 November 2022 is drawing closer. This will provide an excellent opportunity for the public health community to discuss the most pressing public health issues, including the ones identified through EUPHA’s members questionnaire. Droogers, Guichardon and Verschuuren in the EUPHA office column describe the outcomes of this questionnaire. According to EUPHA’s members, mental health is a top priority. Kluge *et al*. describe how WHO is supporting Ukraine in helping their population cope with the mental health impacts of the war. Next to mental health, EUPHA’s members identified lifestyle factors and related chronic conditions, such as cancer, cardiovascular disease and diabetes, as important priorities. Cancer is also a main area of attention in European Union (EU) health policy, with Europe’s Beating Cancer Plan at the heart of the action. Kyriakides describes the latest EU policy developments related to cancer screening. And although the Berlin conference still has to take place, preparations for the 2023 conference in Dublin are already well underway. Staines and Barnhoorn invite you to this 16th EPH conference and explain the conference theme.

## EUPHA office column: our members’ public health priorities for the coming 5 years

DroogersMaaike(EUPHA)GuichardonMarie(EUPHA)VerschuurenMarieke(EUPHA)

What do EUPHA members perceive to be the most important priorities for public health? We ask this question to our members once every 5 years. The most recent members questionnaire from 2021 shows that our members consider mental health to be the top one priority. In addition, lifestyle factors are key as they underly many chronic conditions that are deemed important priorities, such as cancer, cardiovascular disease and diabetes.

Next to general priorities, we asked for priorities for men and women and for specific age groups. Respondents were instructed to exclude COVID-19, as this is a priority in every country. The respondents indicated that the most important priority topics for women are cancer and reproductive health, while for men these are alcohol use, tobacco smoking and cardiovascular diseases. For youth, overweight and obesity, physical inactivity, substance abuse (including smoking and alcohol) and e-addiction and cyber bullying were mentioned as important areas of attention. Inequalities including social inequalities were identified as the main challenge for adults, and cognitive decline and dementia for older people.

The questionnaire was distributed among EUPHA’s Governing Board members representing our full members (i.e. national public health associations) and institutional members as well as several WHO Country Offices in those countries in the WHO European region in which EUPHA does not have a member. We received responses from 56 individuals from 35 countries. 26 came from professionals affiliated with a national public health association, and 18 from professionals affiliated with a public health institute. More information on the methods and the outcomes of EUPHA’s members questionnaire can be found in an infographic that is available at the EUPHA website.^1^

Having these 5-yearly check-ins with our members is very valuable for EUPHA, as it gives us guidance for our activities. We use these outcomes to guide us to focus our advocacy, getting and keeping these public health issues on the political agendas, both at national and EU level. One advocacy opportunity for example is the new thematic network of the European Commission’s Health Policy Platform on Mental Health in All Policies. EUPHA will be involved in this network through the EUPHA Public Mental Health section. We also use the outcomes for guidance for the daily themes of the next editions of the yearly European Public Health Week. Other tools through which we raise awareness for and work on the priorities identified in the questionnaire are e-collections and supplements in the European Journal of Public Health, and dedicated sessions during the annual European Public Health Conference. In the previous conference for example, which took place last month in Berlin, there were tracks on health determinants and health inequalities, chronic diseases, and mental health.^2^ Last but not least, we will use the outcomes of the questionnaire to inform the development of new materials for our resources module. This is a part of EUPHA’s website that is password-protected, where we provide capacity and skills building tools and facilitate the exchange of experiences between members. The resources module is an important element of the members benefits at EUPHA.

## A new era for EU cancer screening: another milestone in Europe’s Beating Cancer Plan

KyriakidesStella
(European Commission)

Embarking on any project brings with it many feelings, notably the hope of at what can be achieved and the difference it can make to people’s lives. We have been working together for decades to tackle cancer and our efforts have saved and prolonged lives across the Union. But we need to do more. In 2020, an estimated 2.7 million people in the EU were diagnosed with cancer and 1.3 million persons lost their lives to it. Estimates show that one in two EU citizens will develop cancer during their lifetime. Cancer is currently the second cause of death in the EU, accounting for over 25% of deaths. If we do not reverse these trends, by 2035 cancer will be the leading cause of death in the EU.

This is why, when we presented Europe’s Beating Cancer Plan in February 2021, one of our main aspirations was to put forward an EU Cancer Screening Scheme with a clear ambition: to save and improve quality of life. We know that early diagnosis saves lives and for this, we need optimal screening programmes. Twenty years have passed since EU screening recommendations were adopted in 2003. Medicine and technology have made incredible advances since then. With our new EU recommendations presented in September, we are marking a turning point for cancer screening in the EU, based on the most recent scientific evidence, as well as two fundamental principles: equal access and quality control of screening. It is unacceptable that, for example, the coverage of the target population ranges from 6% to 90% for breast cancer screening and from 20% to 70% for colorectal cancer screening.

Through the Recommendation, we are aiming to deliver on our ambitious commitment under the Cancer plan to ensure that 90% of people in the EU who qualify for breast, cervical and colorectal screenings are offered such screening by 2025. To do this, we will update the target population and the tests to be used for breast, cervical and colorectal cancer screening. In addition, we are recommending to Member States to extend organized screenings to include also lung, prostate, and gastric cancers—additional cancer sites currently not part of the 2003 recommendation and for which we now have evidence that with screening we can ensure earlier diagnosis, reduce cancer deaths and improve quality of life of patients. With these updates, we are covering six cancer sites that together account for almost 55% of all new cancer estimated to be diagnosed in the EU every year and which cause over 50% of deaths related to cancer.

It is clear that quality controlled population-based screening leads to better public health which in turn will ease the burden of cancer on society across Europe. To support our commitment, we have the means available, and it is now a matter of using these resources to achieve our goals. With unpreceded funding of €4 billion for the Cancer Plan, including around €140 million for better screening alone. We know that an investment in stronger public health is priceless. The cost of not making such an investment now is much greater. At present, the toll that cancer care places on the EU economy is over €100 billion per year. Our cancer screening recommendation will also save millions of euros that can otherwise go into other life-changing projects across health systems in the EU.

With Europe’s Beating Cancer Plan, for the first time, we have an ambitious and well-funded plan at EU level that allows us to take decisive action to change the realities for cancer patients, families and our societies. We can be proud of what we have all achieved together and are determined to continue doing more to change the realities of cancer, as we take another step towards an ever-stronger European Health Union.

## Responding to the mental health impact of the war in Ukraine

KlugeHans Henri P
(WHO Regional Office for Europe)
HabichtJarno
(WHO Ukraine)
LazeriLedia
(WHO Regional Office for Europe)
Ladyk-BryzgalovaAlisa
(WHO Ukraine)
WadiaRoy
(WHO Regional Office for Europe)
Sabet-ParryRayyan
(WHO Ukraine)
RoshchenkoAlona
(WHO Ukraine)
MaurerJason
(WHO Regional Office for Europe)

As we write this, at almost the 8-month mark of the war in Ukraine, WHO has confirmed well over 620 attacks on health wherein hospitals and other critical infrastructure have been damaged or destroyed. Amid the recent escalation in fighting witnessed October 10 onwards, the figure will likely climb even higher. But buildings can be repaired or rebuilt. Ambulances and oxygen tanks can be replaced. Medical supplies can be replenished. The human toll, though, is far greater. More than 6300 civilians—healthcare workers among them—have been killed. About 7 million people are internally displaced, another 7.4 million are currently living as refugees in surrounding countries and beyond. And possibly the most damaging legacy of the war—its impact on mental health on a scale unprecedented in Europe since the end of World War II. Globally, it’s estimated that around one in five people in conflict settings have a mental health condition. The situation in Ukraine is no exception. About 9.6 million people—an estimated 22% of the population currently living in areas affected by conflict—will, at any time during the next 10-year period, likely have some form of mental health challenge. One in 10 of those will experience a moderate or severe condition like depression with suicidal behaviour or psychosis. People with pre-existing mental health conditions who relied on public mental health and social care are facing additional challenges in accessing the services they need. The greatest needs are in areas the most severely impacted by the conflict, but people across the country are also affected by anxiety or sadness, difficulty sleeping, fatigue, anger and unexplained somatic symptoms. These are all normal reactions to abnormal situations and for most people these symptoms do improve over time, especially if they can meet their basic needs and access social support—a challenge at this time.

Even before the war, Ukraine had embarked on an ambitious health reform process, including efforts to strengthen mental health services. This foundation has by and large enabled the wider mental health system to respond fairly quickly to the ongoing emergency. But the system is hard pressed to meet rising demand. Realizing this, the government—together with over 200 partners on the ground—has rapidly stepped up investments in, and delivery of, mental health and psychosocial support, both on the national and, most important, local or community level. In the context of the ongoing war, the people of Ukraine have so far shown a high level of resilience. Yet, they need support in learning to manage their own stress and support each other, while also ensuring that those who develop mental health conditions can readily access services that are safe and evidence-based. Psychological interventions and clinical management of mental health conditions should be scaled up. The Mental Health Gap Action Programme (mhGAP) establishes clinical protocols for non-specialist settings such as primary health care, allowing family doctors and nurses to identify and manage common mental health conditions, including stress-related ones. For more severe cases, Community Mental Health Teams established in Ukraine in 2016, amid earlier conflict, are proving their value all the more. For the diaspora of Ukrainian refugees in various countries, mental health services are provided in partnership with host governments and humanitarian aid partners. And, not least, a crucial component of mental health interventions is the well-being of front-line responders—as Ukraine’s health and social care providers after suffering burnout battling the COVID-19 pandemic are now tasked with responding to the war.

It may seem difficult to snatch good news out of the grim headlines from Ukraine. But it is genuinely encouraging, even inspiring, to see how resilient the health system has proven all this while, constantly tested yet standing—including the focus on mental health, an issue that all too often is neglected, even during crises. Ukraine has set an example for the entire WHO European Region on how the Government, under the auspices of the First Lady of Ukraine and in coordination with partners, has responded to the mental health needs of the population. Our appreciation must also be extended to the countless civil society organizations and humanitarian partners providing mental health support across the country, including in newly accessible areas.

Mental health is a flagship initiative of the European Programme of Work 2020–2025 that guides the work of the WHO Regional Office for Europe, endorsed by all 53 of our Member States including Ukraine. The WHO-convened Pan-European Mental Health Coalition is a partnership built on the sharing of knowledge and expertise to improve mental health systems across the Region. By gathering national leaders, professionals, members of civil society, representatives of international organizations and people living with mental health conditions, the Coalition aims to mainstream mental health across all sectors of society and ensure that everybody can access resources needed for good mental health. Its key priorities, stemming from the European Framework for Action on Mental Health (2021–2025) are to transform mental health services, integrate mental health into emergency response and recovery efforts, and promote mental health and prevent mental ill health across the life-course. The Coalition is actively drawing on lessons learnt from the war in Ukraine to support other countries in developing their own emergency response measures.

Ukraine is determined not only to restore its damaged health mechanisms and infrastructure, but to do so smarter and better. Within this herculean effort, the building of the mental health system in Ukraine will continue both despite, and because of, the war. The Ukrainian government and people know full well, more than ever, how critical mental health is for individual and national recovery—including the wellbeing of future generations. We are determined to help them succeed.

*Hans Henri P. Kluge (WHO Regional Office for Europe), Jarno Habicht (WHO Ukraine), Ledia Lazeri (WHO Regional Office for Europe), Alisa Ladyk-Bryzgalova (WHO Ukraine), Roy Wadia (WHO Regional Office for Europe), Rayyan Sabet-Parry (WHO Ukraine), Alona Roshchenko (WHO Ukraine), Jason Maurer (WHO Regional Office for Europe)*

## 16th European Public Health Conference, 8–11 November 2023, CCD, Dublin, Ireland

StainesAnthony(EPH Conference)BarnhoornFloris(EPH Conference)

### Welcome to Dublin

We originally planned this EPH Conference for November 2021, but the COVID-19 pandemic dictated otherwise. We ended up with a successful virtual conference in 2021 but in November 2023, we will be back in person, in Dublin.

Over the next decade, we will all make choices which will determine the future of our advanced technological society. The COVID-19 and monkeypox pandemics, through which the world is now living, are as graphic an example as could be desired of the instability of our model of life, and the need for action on One Health. In Europe, we have paid a high price for our belief that we were safe from infection, that we could dismantle our public health systems, and get away with it. Other countries to whom COVID-19 spread from Europe have suffered more, and paid even higher prices.

We have now run out of road on climate emergency. 2021 was the warmest year on record, and 2022 and 2023 are likely to beat that record. The global climate is changing rapidly. Building a future, any kind of future, for us and for our children, demands a new attention to sustainability. It’s tempting to despair, to give up. Let’s not.

This conference hopes to open up part of this discussion, with a focus on health, and health care. We will look specifically at gender and health, at healthcare delivery generally, the use of digital tools, and the necessary staffing and skills to provide good care. We will, in line with the overall conference theme, look in depth at the climate emergency, and very specifically at the human food supply. Russia’s invasion of Ukraine poses serious risks to global food security that will require a range of responses from governments and international organizations. The unfolding crisis in Ukraine will push up already-high food price inflation, and have serious consequences for low-income net-food importing countries, many of which have seen an increase in malnourishment rates over the past few years in the face of pandemic disruptions. The Ukraine conflict not only exposes the ruthlessness of the current Russian government but also our failure to create a resilient sustainable global food supply. We can do better, and we have to do better.

We look forward to seeing you all in person, in Dublin. Dublin is well known as a literary city, but our long history of public health in Ireland is less well known. These two elements of our history intersect strikingly in one man, William Wilde, society doctor, hospital founder, apprentice to Abraham Colles, husband to Jane Elgee, herself better known as ‘Speranza’, the poet of revolution, father to Oscar Wilde, who needs no introduction, and Willy, an alcoholic and a journalist. Sir William, as he became, wrote extensively on eye surgery, Irish antiquities, Irish folklore, and on the health of the Irish population. You may yet have a chance to discover his city, in all its brightness and darkness, as well as learning something more about making and sustaining our futures.

The main theme of the 16th EPH Conference is: ‘Our Food, Our Health, Our Earth: A Sustainable Future for Humanity’. Subthemes are:

- Food policy, global health and the SDGs
- Gender health at the margins or the centre?
- Digital Health
- Workforce and Skills
- Climate Emergency

### Abstract submission for Dublin 2023

Participate at Dublin 2023 by sharing your work with colleagues. The EPH Conference offers excellent opportunities to learn from the latest research and practice, to network with experts and colleagues and to expand your professional horizons. Abstracts are invited for workshops and single presentations in all areas of public health research, practice, policy and education. Abstract submission is open from 1 February to 1 May 2023. Abstracts are considered for workshops, oral, pitch and poster presentations. Do not miss the opportunity to share your work at the largest public health event in Europe. For less experienced abstract submitters a mentor programme is set-up. Be recognized for your hard work. Detailed instructions can be found on http://www.ephconference.eu.
